# Left Atrial Appendage Resection for Prevention of Systemic Embolism - New Scientific Evidence

**DOI:** 10.21470/1678-9741-2021-0958

**Published:** 2021

**Authors:** Renato A. K. Kalil, Leandro I. Zimerman, Álvaro Avezum

**Affiliations:** 1 Department of Surgery, Universidade Federal de Ciências da Saúde de Porto Alegre, Porto Alegre, Rio Grande do Sul, Brazil.; 2 Post-Graduation Program, Fundação Universitária de Cardiologia, Porto Alegre, Rio Grande do Sul, Brazil.; 3 Department of Internal Medicine, Universidade Federal do Rio Grande do Sul, Porto Alegre, Rio Grande do Sul, Brazil.; 4 Sociedade Brasileira de Arritmias Cardíacas, São Paulo, São Paulo, Brazil.; 5 Sociedade de Cardiologia do Estado do Rio Grande do Sul, Porto Alegre, Rio Grande do Sul, Brazil.; 6 International Research Center, Hospital Alemão Oswaldo Cruz, São Paulo, São Paulo, Brazil.; 7 Cardiopneumology Department, Faculdade de Medicina, Universidade de São Paulo, São Paulo, São Paulo, Brazil.; 8 Population Health Research Institute, McMaster University, Ontario, Canada.; 9 National Coordinator, Left Atrial Appendage Occlusion Study (LAAOS III), São Paulo, São Paulo, Brazil.

The left atrial appendage (LAA) has been proven to be a source of cerebral and systemic emboli, especially in cases of paroxysmal or permanent atrial fibrillation (AF) and in association with structural heart disease. LAA has variable morphology, which leads to a higher risk of thromboembolism - in the abovementioned circumstances, the greater the risk the higher the patient’s risk index, calculated by the CHA_2_DS_2_VASc score^(1)^.

In patients undergoing cardiac surgery for other indications, LAA resection or exclusion has been proposed to prevent immediate or late postoperative embolisms. Even in patients without a history of AF, the incidence of paroxysmal immediate postoperative AF is 30 to 50% of operated adults^(2)^. In these cases, systemic embolization is not infrequent and eventually occurs if AF is not reversed or anticoagulation is started. There are suggestions in the literature for LAA resection whenever the chest is opened for cardiac surgery, which lacks an assessment of the risk/benefit balance^(3)^.

However, in patients who have already had episodes of paroxysmal AF or are in permanent AF and are undergoing coronary artery bypass graft surgery, valve repair or replacement, and aortic surgery, a consensus has been formed that LAA resection could be potentially beneficial. Resection and suture is the method that ensures the success of the procedure. Other methods of surgical exclusion might be associated with the persistence of residual communication between the LAA and the atrial cavity^(4)^. When the resection is not performed, the exclusion can be done externally by a special clip or by a stapler and must be verified by transesophageal echocardiography (TEE).

The thromboembolism prevention hypothesis in this particular clinical setting was recently evaluated and reliably confirmed with the presentation of the results of the Left Atrial Appendage Occlusion Study (LAAOS III) randomized clinical trial^(5)^. In this trial, adult patients, candidates for cardiac surgery, with a history of AF and a CHA_2_DS_2_VASc score ≥ 2 were randomized to LAA resection or not during surgery. Three forms of LAA resection/exclusion were accepted: surgical resection, suture, or exclusion by applying a special click or using a stapler. A fourth form was accepted in patients undergoing surgery by video-assisted right mini-thoracotomy, the double internal running suture, confirmed by TEE.

In the LAAOS III trial, carried out with the sponsorship from both a research funding academic organization (the Population Health Research Institute) and a research funding agency (the Canadian Institutes of Health Research), 4,811 patients, mean CHA_2_DS_2_VASc score of 4.2, in 105 centers in 27 countries, were included and randomized 1:1 for LAA occlusion or not, maintaining the recommendation for anticoagulation in all. Due to the strength of the benefits from the procedure, the trial was stopped early. In a mean follow-up of 3.8 years, the reduction in the risk of the stroke/systemic embolism (S/SE) outcome was 33% (HR=0.67 [95% CI 0.53-0.85]; P=0.001) in total and 42% if only the late period was computed, after the 30^th^ postoperative day (exploratory landmark analysis). The benefits amplified progressively overtime. Importantly, 77% of patients were on anticoagulants within the three-year follow up. From a clinical impact standpoint, the number needed to treat was 42, which means, for each 42 patients undergoing LAA occlusion, one prevents one case of S/SE. Computing only ischemic stroke, the relative risk reduction was 34%. Benefits were consistent across all subgroups, such as age, gender, geographic location, type of AF, hypertension, valve surgery, ventricular function, thromboembolic risk score, rheumatic disease, previous history of cerebral ischemia, and type of anticoagulation. Interestingly, mortality and the incidence of systemic, non-cerebral embolism were similar between groups, unlike cerebral ischemia. The incidence of perioperative bleeding, heart failure, or death did not differ significantly between the trial groups.

This trial had significant global impact. It was published concomitantly to the presentation at the American College of Cardiology (ACC) annual session^(5)^. In an editorial in the same issue^(6)^, Richard Page comments on the relevance and power of the evidence found and predicts that the exclusion of LAA, in the population studied, should receive a level 1 recommendation in future guidelines. Important comments were also brought by John Mandrola in Dip Dive Discussion, still at ACC 2021, and on the Medscape website^(7)^, where it was highlighted that the studied population continued to use anticoagulants.

LAA resection or occlusion, however, are still not incorporated in routine surgical practice, except as part of arrhythmia surgery^(8)^. The subject of S/SE prevention by LAA resection has been explored from long time, associated to AF surgery^(9)^, but never before had been demonstrated with such power. The additional benefit of excluding LAA in anticoagulated patients can be better understood with a recent paper that shows that LA thrombus prevalence is high in subgroups of anticoagulated patients with AF, who may benefit from routine pre-procedural TEE use before cardioversion or catheter ablation^(10)^. Under available data, it could not yet be said that LAA exclusion can dispense anticoagulation, although approximately 25% of LAAOS III patients have stopped taking anticoagulant in the postoperative follow-up. Another scientific question would be on the benefit of excluding the LAA in the population who abandoned anticoagulation.

The study has great clinical relevance and internal and external validity, as it included typical adult cardiac surgery patients in centers around the globe. It had answered a clear question, reliably and through “hard” outcomes, minimal loss of follow-up, and the intention-to-treat analysis is consistent with as-treated analysis. The procedure is easily reproducible in any cardiovascular surgery center. The exclusion of the LAA is a simple and quick procedure, with minimal complications. In the study, it represented an average increase of six minutes of cardiopulmonary bypass and four minutes of ischemic heart arrest times, with no differences in postoperative bleeding, heart failure, and rate of reoperations.

In summary, the LAAOS III trial demonstrated that the exclusion of LAA in the adult population undergoing cardiac surgery, with a history of AF and a CHA_2_DS_2_VASc score ≥ 2, adds long-term benefit in reducing embolic events, maintained the recommendation for anticoagulation. Whether these patients will be able to dispense the anticoagulation therapy is a question to be further investigated. Consequently, LAAOS III results should not be extended to percutaneous LAA occlusion. This question had been endorsed in the Medscape site commentator’s opinion^(7)^: “LAAOS III studied appendage closure ‘in addition to’ standard care, including anticoagulation. Percutaneous closure trials studied an entirely different strategy: that is, after a period of time, presumably with endothelization of the device, the goal is to stop anticoagulation. In studies comparing the Watchman implantable device against warfarin, ischemic strokes were higher in the Watchman arm; therefore, the benefit (if any) from percutaneous closure must come from reducing the burden (major bleeding) of long-term use of anticoagulation. Another reason LAAOS III does not inform the percutaneous device-based strategy is that direct surgical closure will certainly be more complete than that achieved by endocardial devices, given the highly diverse appendage anatomy. Incomplete percutaneous closure, reported in nearly a third of patients implanted with the Watchman device in the PROTECT AF trial, is a major problem because it reduces the probability of stopping anticoagulation and may increase the risk for ischemic stroke.” The eventual benefit of adding LAA percutaneous closure to anticoagulation in high-risk patients to decrease embolic events is a strategy that needs to be studied.

In conclusion, it can be stated that exclusion by resection and suture, or by clip application or stapler application, should be incorporated into the routine cardiac surgery practice in adult patients with a history of AF and CHA_2_DS_2_VASc score ≥ 2. Surgeons, hospitals, and healthcare providers must be prepared to include this evidence-based strategy in their centers and clarify the expected benefit to their patients.
